# Burden of bloodstream infection in an area of Mid-Norway 2002-2013: a prospective population-based observational study

**DOI:** 10.1186/s12879-017-2291-2

**Published:** 2017-03-11

**Authors:** Arne Mehl, Bjørn Olav Åsvold, Stian Lydersen, Julie Paulsen, Erik Solligård, Jan Kristian Damås, Stig Harthug, Tom-Harald Edna

**Affiliations:** 1 Department of Medicine, Levanger Hospital, Nord-Trøndelag Hospital Trust, post box 333, Levanger, N-7601 Norway; 2 Unit for Applied Clinical Research, Department of Cancer Research and Molecular Medicine, Norwegian University of Science and Technology, Trondheim, Norway; 3 Mid-Norway Sepsis Research Group, Faculty of Medicine, NTNU, Trondheim, Norway; 4 Department of Public Health and General Practice, Norwegian University of Science and Technology, Trondheim, Norway; 5 Department of Endocrinology, St Olav’s Hospital, Trondheim University Hospital, Trondheim, Norway; 6 Regional Centre for Child and Youth Mental Health and Child Welfare – Central Norway, Norwegian University of Science and Technology, Trondheim, Norway; 7 Centre of Molecular Inflammation Research, Department of Cancer Research and Molecular Medicine, Norwegian University of Science and Technology, Trondheim, Norway; 8 Clinic of Anaesthesia and Intensive Care, St Olav’s Hospital, Trondheim University Hospital, Trondheim, Norway; 9 Department of Circulation and Medical Imaging, Norwegian University of Science and Technology, Trondheim, Norway; 10 Department of Infectious Diseases, St Olav’s Hospital, Trondheim University Hospital, Trondheim, Norway; 11 Department of Research and Development, Haukeland University Hospital, Bergen, Norway; 12 Department of Clinical Science, University of Bergen, Bergen, Norway; 13 Department of Surgery, Levanger Hospital, Nord-Trøndelag Hospital Trust, Levanger, Norway

**Keywords:** Bloodstream infection, Bacteremia, Bacteraemia, Sepsis, Population-based, Incidence, Mortality, Case fatality

## Abstract

**Background:**

Studies from several countries indicate that the incidence and mortality of bloodstream infection (BSI) have been increasing over time.

**Methods:**

We studied the burden of disease and death related to BSI in a defined geographical area of Mid-Norway, where BSI episodes were prospectively recorded by the same microbiological department during 12 consecutive years. Death from BSI was defined as death within 30 days of BSI detection. Age and sex standardized incidence and mortality rates and case fatality rates were calculated.

**Results:**

Between 2002 and 2013, 1995 episodes of BSI in 1719 patients aged 16 to 99 years were included. The overall incidence of BSI was 215 per 100,000 person-years. The incidence increased exponentially with age, particularly in males. The incidence increased from 205 to 223 per 100,000 person-years from 2002–07 to 2008–13. *Escherichia coli* was the most frequently isolated infective agent, followed by *Streptococcus pneumoniae* and *Staphylococcus aureus*. The rate of *S. pneumoniae* BSI decreased over time in males (on average by 9.2% annually), but not in females. The total rate of BSI microbes with acquired resistance increased slightly over time, but did not exceed 2 episodes per 100,000 person-years. The mortality of BSI was 32 per 100,000 person-years, higher in males than in females (36 vs. 28 per 100,000 person-years) and was significantly higher in old age, particularly in males. The total BSI mortality was similar in the first and second halves of the study period, but the mortality of *S. pneumoniae* BSI decreased in males (15.0% annually). The crude case fatality decreased from the first to the second half of the study period (17.2% to 13.1%; *p =* 0.014). The rate of blood culture sampling increased more than twofold during the study period.

**Conclusions:**

The mortality of BSI remained stable during 2002–2013. At the same time, BSI incidence increased and case fatality rate decreased, perhaps because an increased rate of blood culture sampling may have led to improved detection of milder BSI episodes. Very low, yet slightly increasing rates of microbes with acquired resistance were observed.

**Electronic supplementary material:**

The online version of this article (doi:10.1186/s12879-017-2291-2) contains supplementary material, which is available to authorized users.

## Background

Bloodstream infection (BSI) contributes substantially to morbidity and mortality worldwide [1]. The annual incidence has been reported between 80 and 257 per 100,000 person-years [2–9]. In Europe, the annual number of BSI episodes and deaths associated with BSI has been estimated at 1.2 million and 157,000, respectively. The corresponding numbers of hospital-acquired BSIs were found to be 240,000 episodes and 29,000 deaths [1]. Most studies report increasing incidence rates [5, 6, 8], but a decreasing rate has also been described [10].

The burden of BSI includes mortality as well as incidence, but few studies have reported both [1, 3, 6]. Monitoring the burden of BSI is important for reasons of resource allocation and for evaluating prevention and treatment strategies [11]. As the proportion of elderly people, more prone to infections, is increasing [12, 13], knowledge about their burden of severe infections is of particular importance. Growing antimicrobial resistance worldwide, associated with increased mortality [14, 15], make surveillance of BSI microbes and antimicrobial resistance essential. As different prevention and treatment strategies are needed (vaccination programs, antibiotic regimens, and infection control measures), it is necessary to separately survey community acquired, health care-associated, and hospital acquired BSIs [16, 17].

We conducted a prospective study within an area of Mid-Norway to assess the BSI incidence and mortality, with emphasis on age and sex differences and time trends.

## Methods

As part of the Mid-Norway Sepsis Study we prospectively recorded episodes of BSI in patients aged 16 years or older admitted to Levanger Hospital between January 1, 2002 and December 31, 2013. This BSI cohort or parts of it have previously been used in studies on other aspects of BSI [18–20], and two studies, describing BSIs with *Staphylococcus aureus* 1995–2011 [21] and *Streptococcus pneumoniae* 1993–2011 [22], have included the respective bacterial species from the current cohort. Levanger Hospital is one of two hospitals in Nord-Trøndelag County. The hospital is an emergency hospital serving the population in a defined geographical area of 10 municipalities, with 68,491 inhabitants aged 16 years and above at the start of the study, and 75,858 at the end of the study. Population data of the ten municipalities around Levanger Hospital for every year between 2002 and 2013, with age and sex distribution, was obtained from Statistics Norway.

The microbiology laboratory at Levanger Hospital exclusively provided all microbiology services in Nord-Trøndelag County. Patients hospitalized with BSI were identified and prospectively registered at the microbiology laboratory. For each BSI, a registration form was completed by the responsible physician. The main investigator, two subordinate doctors and three research nurses reviewed the patients’ records to verify the data and record additional variables. Blood cultures were performed in BACTEC 9240 Vacutainer Culture Bottles (Becton Dickinson Diagnostic Instrument Systems, Sparks, MD) [23], which in 2010 was replaced by BACTEC FX. The volume of blood drawn was the same during the study period. A blood culture set consisted of one aerobic and one anaerobic BACTEC bottle obtained from a single draw. If a second draw was taken simultaneously from another site, one aerobic bottle was used. Isolates were identified using standard methods [24]. Antimicrobial susceptibility testing was performed by the disc diffusion method (Neo-Sensitabs, Rosco Diagnostica, Taastrup, Denmark). The microbiology laboratory at Levanger Hospital is ISO 15189 accredited.

An episode of BSI was defined by growth of one or more microbes from blood culture combined with clinical evidence of systemic infection [25]. A new BSI episode with the same microbe in the same patient was recorded if an interval of at least 30 days had passed without signs of infection since an earlier episode [26]. If more than one organism was isolated from one or more blood cultures within a 72-h period, the BSI episode was classified as polymicrobial. One positive blood culture for organisms regarded as etiological agents was the requirement for inclusion. For coagulase-negative staphylococci, alpha-hemolytic streptococci, or other possible skin contaminants, at least two identical isolates from separate venipunctures were required.

The BSI episodes were classified as hospital-acquired (HA), health care-associated (HCA) or community acquired (CA) [16, 17]. HA-BSI was diagnosed if the infection was detected >48 h after admission [27]. Patients who during the 30 days prior to hospital admission had (1) been hospitalized two or more days or (2) had received intravenous therapy or wound care at home or (3) hemodialysis or chemotherapy at hospital visits or (4) were nursing home residents, were categorized as having HCA-BSI. CA-BSI was diagnosed if the infection was detected <48 h after admission and none of the criteria for HCA-BSI were fulfilled.

We defined death from BSI as death within 30 days of BSI detection. The patient administrative system at the hospital receives updated information on vital status from the national population register, and thus, information on fatal outcome of BSI was complete even if the patient was discharged from hospital.

A urinary focus was assigned when the same microbe was isolated from urine and as well as from blood culture along with clinical signs/symptoms or risk factors for urinary infection, and no other source of infection was identified. A presumed pulmonary focus was diagnosed with clinical signs of lower respiratory infection accompanied by positive radiologic findings. Focus in the biliary tract was ascertained based on clinical, biochemical and radiological findings. Signs of infection along with focal growth of the same microbe as in blood culture were taken as a confirmation of infection in abdomen, skin, soft tissue or other sites. An unknown focus of infection was assigned when none of the criteria for ascertaining a focus were met.

### Statistical analyses

BSI included both first-time and repeat episodes [6, 7]. The mortality rate was calculated as the total number of deaths within 30 days of a diagnosis of BSI per 100,000 person-years. Incidence and mortality rates were calculated for the population between 16 and 99 years. Population data during 2002–13 (Statistics Norway), were used as denominators to calculate age-specific and sex-specific rates of BSI episodes and BSI related deaths. The incidence rate of HA-BSI was also reported as the number of patients with HA-BSI in a time period divided by the number of hospital bed-days in that same time period. Observed incidence and mortality rates were standardized to the age and sex distribution of the population of Norway 2010. Confidence intervals (CIs) of rates were calculated based on assumed Poisson distribution. Poisson regression was used to assess time trends in BSI incidence and mortality rates (average rate ratio per calendar year), adjusted for age (in 5-year intervals) and stratified by sex.

The case fatality rate was defined as the total number of deaths within 30 days of diagnosed BSI episodes divided by the total number of BSI episodes. Confidence intervals were calculated using Wilson’s approximation to the binominal distribution [28]. Case fatality rates for two time periods were compared using chi-square test. Two-sided *p-*values <0.05 were considered significant and 95% confidence intervals (CI) have been reported where relevant. The analyses were performed using SPSS 22, Stata 13, and StatXact 9.

## Results

During the 12-year study period, a total of 1995 episodes of BSI occurred, 1034 in males and 961 in females, among 1719 individuals.

### Incidence rates allover and by sex and age

**Fig. 1:**
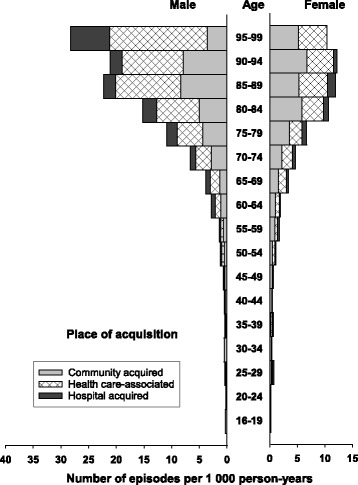
Incidence of bloodstream infection (BSI) by place of acquisition, age, and sex. The figure shows the number of BSI episodes per 1000 person-years. The number multiplied by 100 gives the rate per 100,000 person-years

**Fig. 2:**
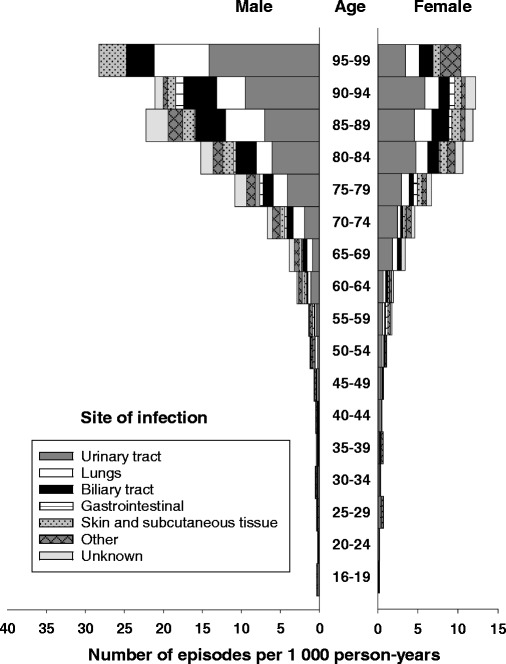
Incidence of bloodstream infection (BSI) by site of infection, age, and sex. Number of BSI episodes per 1000 person-years is shown. The number multiplied by 100 gives the rate per 100,000 person-years

**Fig. 3:**
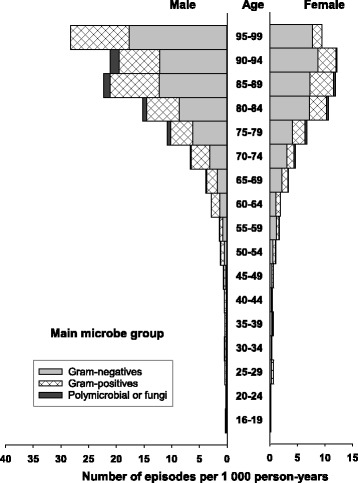
Incidence of bloodstream infection (BSI) by main microbe group, age, and sex. The figure shows the number of BSI episodes per 1000 person-years. The number multiplied by 100 gives the rate per 100,000 person-years

The overall incidence rate of BSI (first-time and repeat episodes) was 215 per 100,000 person-years (Table 1), and the rate was higher in males than in females (222 vs. 209 per 100,000 person-years). Age-specific BSI rates were substantially higher in males than in females, particularly in older age groups (Figs. 1, 2 and 3; Additional file 1: Table S1). Whereas the incidence was similar for males and females 16–64 years (82 vs. 83 per 100,000 person- years), males had substantially higher incidence than females in the age group ≥80 years (1826 vs. 1126 per 100,000 person-years).

**Table 1:** Incidence of bloodstream infection (BSI) stratified by sex in an area of Mid-Norway 2002-2013. Number of episodes and observed and standardized incidence rates, allover and in various subgroups, are shown

	Total			Females			Males		
Group of BSI	*n*	Observed BSI rate^a^ (95% C.I.)	Age and sex standar- dized rate^b^	*n*	Observed BSI rate^a^ (95% CI)	Age standar-dized rate ^c^	*n*	Observed BSI rate^a^ (95% CI)	Age standar-dizedrate ^d^
All BSIs	1995	232 (222–242)	215	961	221 (208–236)	209	1034	242 (228–258)	222
Age^e^									
16–64 years	578	84 (78–92)	82	284	84 (75–94)	83	294	84 (75–95)	82
65–79 years	692	564 (523–607)	555	306	477 (425–534)	473	386	658 (594–727)	646
≥80 years	725	1379 (1280–1483)	1373	371	1122 (1011–1242)	1126	354	1813 (1629–2012)	1826
Place of acquisition									
CA-BSI	934	109 (102–116)	102	502	116 (106–126)	110	432	101 (92–111)	94
HCA-BSI	787	91 (85–98)	85	356	82 (74–91)	78	431	101 (92–111)	91
HA-BSI	274	32 (28–36)	30	103	24 (19–29)	23	171	40 (34–47)	37
Infection focus									
Urinary tract	752	87 (81–94)	81	429	99 (90–109)	94	323	76 (68–84)	68
Lungs	331	38 (34–43)	36	142	33 (28–39)	31	189	44 (38–51)	41
Biliary tract	220	26 (22–29)	24	93	21 (17–26)	21	127	30 (25–35)	27
Gastrointestinal tract	101	12 (10–14)	11	44	10 (7–14)	9.8	57	13 (10–17)	13
Skin or soft tissue	143	17 (14–20)	16	61	14 (11–18)	13	82	19 (15–24)	18
Other	247	29 (25–33)	27	104	24 (20–29)	23	143	34 (28–39)	31
Unknown	201	23 (20–27)	22	88	20 (16–25)	19	113	26 (22–32)	24
Microbe group									
Gram-negative BSI	1133	132 (124–140)	123	616	142 (131–154)	135	517	121 (111–132)	110
Gram-positive BSI	777	90 (84 to 97)	85	310	71 (64–80)	68	467	109 (100–120)	101
Polymicrobial or fungal BSI	85	10 (8–12)	9	35	8 (6–11)	8	50	12 (8–15)	11
Microbes (the four most common)									
*Escherichia coli*	686	80 (74–86)	74	421	97 (88–107)	93	265	62 (55–70)	56
*Streptococcus* *pneumoniae*	226	26 (23–30)	25	109	25 (21–30)	24	117	27 (23–33)	25
*Staphylococcus* *aureus*	218	25 (22–29)	24	75	17 (14–22)	16	143	34 (28–39)	31
*Klebsiella* spp*.*	134	16 (13–18)	14	65	15 (12–19)	14	69	16 (13–20)	15

^a^BSI episodes per 100,000 person-years (totally 860,630 person-years in individuals ≥16 years, 426, 517 in males, 434,113 in females)

^b^males and females standardized to the age distribution of the male and female population of Norway 2010, respectively

^c^standardized to the age distribution of females in Norway 2010

^d^standardized to the age distribution of males in Norway 2010

^e^Person-years in the three age groups were: 16–64 years: males 348,342; females 336,950; 65–79 years: males 58,645; females 64,100; ≥80 years: males 19,530; females 33,063

### Place of acquisition

The rates of CA-, HCA-, and HA-BSI were 102, 85, and 30 per 100,000 person-years, respectively (Table 1). HCA- and HA-BSIs constituted larger parts of the BSIs in men than in women, and HCA- and HA-BSI predominated among men in the oldest age groups (Fig. 1). The total rate of HA-BSI was 38 per 100,000 hospital bed-days (Additional file 1: Table S2).

### Site of infection

The urinary tract was the predominant site of infection (no. of episodes per 100,000 person-years: 81 overall, 94 in females and 68 in males) (Table 1), followed by the lungs and the biliary tract. Rates of BSI from the urinary tract, lungs, and biliary tract were higher in old age, particularly in males (Fig. 2).

### Microbes

Gram-negative BSI was more common in females than in males (135 vs. 110 per 100,000 person-years) (Table 1), whereas Gram-positive BSI most often occurred in males (101 vs. 68 per 100,000 person-years). *Escherichia coli* was the most commonly isolated BSI microbe from all three places of acquisition and predominated in females (93 vs. 56 per 100,000 person-years) (Table 1). The second most common microbe was *Streptococcus pneumoniae* (25 episodes per 100,000 person-years), which was evenly distributed between the sexes and mainly occurred in CA-BSI. *Staphylococcus aureus*, close to *S. pneumoniae* in total rate (24 per 100,000 person-years), was mostly represented in HCA-BSI and was significantly more frequent in males than in females (31 vs. 16 episodes per 100,000 person-years).

### Time trends in incidence

**Fig. 4:**
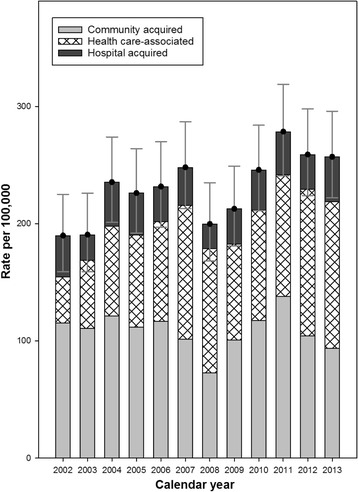
Observed incidence of bloodstream infection (BSI) per calendar year by different places of acquisition. Number of BSI episodes per 100,000 person-years is shown

The observed incidence rate of BSI increased from 190 per 100,000 person-years in 2002 to 257 in 2013 (with a peak at 278 in 2011) (Fig. 4; Additional file 1: Table S3). Standardized incidence rates for two time periods, 2002-2007 and 2008–2013, are shown in Table 2, and age-adjusted time trends in BSI rate by sex are shown in Table 3. Overall, the incidence rate increased from 205 to 223 per 100,000 person-years from 2002–07 to 2008–13. The incidence rate increased on average by 2.8% annually in females but not significantly in males. HCA-BSIs increased from 72 to 96 per 100,000 person-years from 2002–07 to 2008–13. The HA-BSI rate, calculated as episodes per 100,000 hospital bed-days, increased from 36 to 40 (Additional file 1: Table S4).

**Table 2:** Incidence of bloodstream infection (BSI) in two time periods in an area of Mid-Norway. Number of episodes and observed and standardized incidence rates are shown

	2002–2007			2008–2013		
Group of BSI	*n*	Observed incidence rate^a^ (95% CI)	Age and sex standardized incidence rate^b^	*n*	Observed incidence rate^a^ (95% CI)	Age and sex standardized incidence rate^b^
All BSIs	921	221(206–235)	205	1074	242 (228–257)	223
Place of acquisition						
CA-BSI	471	113 (103–123)	106	463	105 (95–115)	98
HCA-BSI	316	76 (68–84)	72	471	106 (97–116)	96
HA-BSI	134	32 (27–38)	30	140	32 (27–37)	29
Infection focus						
Urinary tract	316	76 (68–84)	70	436	98 (89–108)	90
Lungs	169	40 (35–47)	38	162	37 (31–43)	34
Biliary tract	106	25 (21–31)	23	114	26 (21–31)	23
Gastrointestinal tract	47	11 (8–15)	11	54	12 (9–16)	12
Skin or soft tissue	81	19 (15–24)	18	62	14 (11–18)	13
Other	108	25 (21–31)	24	139	31 (26–37)	30
Unknown	94	22 (18–28)	21	107	24 (20–29)	22
Microbe group						
Gram-negative BSI	499	119 (109–130)	111	634	143 (132–155)	131
Gram-positive BSI	386	92 (83–102)	87	391	88 (80–97)	81
Polymicrobial or fungal BSI	36	9 (6–12)	8	49	11 (8–15)	10
Microbes (the four most common)^c^						
*Escherichia coli*	303	73 (65–81)	68	383	86 (78–96)	79
*Streptococcus pneumoniae*	127	30 (25–36)	29	99	22 (18–27)	21
*Staphylococcus aureus*	100	24 (19–29	22	118	27 (22–32)	25
*Klebsiella* spp*.*	47	11(8–15)	10	88	20 (16–24)	18

^a^BSI episodes per 100,000 person-years (417,682 person-years in 2002–2007; 442,948 person-years in 2008–2013)

^b^age and sex standardized to the population of Norway 2010

^c^less common BSI microbes are listed in Additional file 1: Table S5

**Table 3:** Age-adjusted time trends in bloodstream infection (BSI) incidence stratified by sex. The table shows BSI rate ratios per calendar year by Poisson regression

	Females		Males	
	BSI rate ratio (95% CI)	*p-*value	BSI rate ratio (95% CI)	*p-*value
BSI, allover	1.028 (1.010–1.047)	0.003	1.011(0.993–1.029)	0.22
Place of acquisition				
Community acquired	0.998 (0.973–1.024)	0.88	0.976 (0.950–1.004)	0.09
Health care-associated	1.080 (1.047–1.114)	<0.001	1.053 (1.024–1.083)	<0.001
Hospital acquired	1.009 (0.954–1.067)	0.76	0.999 (0.956–1.043)	0.96
Infection focus				
Urinary tract	1.056 (1.027–1.086)	<0.001	1.041 (1.008–1.075)	0.013
Lungs	1.021 (0.974–1.071)	0.39	0.969 (0.930–1.010)	0.14
Biliary tract	1.017 (0.959–1.079)	0.57	0.993 (0.944–1.045)	0.80
Gastrointestinal tract	1.017 (0.933–1.108)	0.70	1.052 (0.975–1.136)	0.19
Skin or soft tissue	0.934 (0.868–1.005)	0.069	0.979 (0.920–1.042)	0.51
Other	0.989 (0.935–1.045)	0.69	1.040 (0.991–1.091)	0.11
Unknown	1.043 (0.981–1.109)	0.17	0.987 (0.935–1.041)	0.64
Microbe group				
Gram-negative BSI	1.043 (1.019–1.068)	<0.001	1.020 (0.995–1.046)	0.12
Gram-positive BSI	0.997 (0.966–1.030)	0.87	0.993 (0.968–1.020)	0.62
Polymicrobial or fungal BSI	1.031 (0.936–1.135)	0.54	1.095 (1.007–1.190)	0.034
Microbes (the four most common)				
*Escherichia coli*	1.044 (1.015–1.073)	0.003	1.002 (0.967–1.037)	0.92
*Streptococcus pneumoniae*	1.007 (0.954–1.063)	0.80	0.908 (0.860–0.958)	<0.001
*Staphylococcus aureus*	0.988 (0.926–1.055)	0.73	1.032 (0.984–1.082)	0.20
*Klebsiella* spp*.*	1.136 (1.053–1.224)	0.001	1.094 (1.020–1.175)	0.012

BSIs from the urinary tract increased from 70 to 90 per 100,000 person-years. Gram-negative BSI increased from 111 to 131 per 100,000 person-years, most evident for *E. coli* (68 to 79 per 100,000 person-years) and *Klebsiella* spp*.* (10 to 18 per 100,000 person-years). Polymicrobial or fungal BSI increased from 8 to 10 per 100,000 person-years, whereas *Streptococcus pneumoniae* BSI decreased from 29 to 21 per 100,000 person years. The rate of *Pseudomonas aeruginosa* BSI was fairly stable (6.7 to 5.8 per 100,000 person-years), while the rate of candida BSI increased slightly over time (1.4 to 2.3 in the first and second period, respectively) (Additional file 1: Table S5).

### BSI microbes with acquired drug resistance

The rate of BSI microbes with acquired drug resistance (ADR) was very low but it increased slightly over time in our population (Additional file 1: Table S5). In the first and second time period, the total rates of ADR microbes were 0.6 and 2.0 per 100,000 person-years. Methicillin-resistant *S. aureus* (MRSA) contributed 0 and 0.2 per 100,000 person-years, penicillin-non-susceptible pneumococci (PNSP) 0 and 0.4 per 100,000 person-years, and *Enterobacteriaceae* producing extended spectrum beta-lactamase (ESBL-E) 0.6 and 1.4 per 100,000 person-years in the first and second time period, respectively.

### BSI mortality

**Fig. 5:**
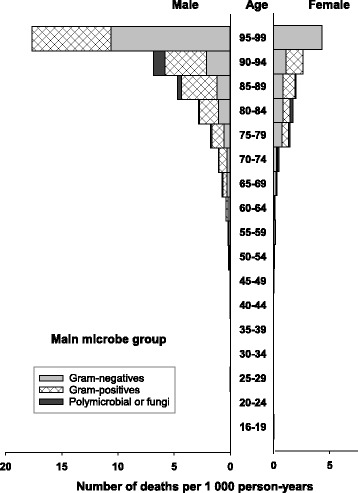
Mortality of bloodstream infection (BSI) by main microbe group, age, and sex. The figure shows number of deaths within 30 days of BSI episodes per 1000 person-years. The number multiplied by 100 gives the rate per 100,000 person-years

Death within 30 days occurred in 299 of the BSI episodes, 172 in males and 127 in females. The overall mortality rate of BSI was 32 per 100,000 person-years (Table 4). The mortality rate was higher in males than in females (36 vs. 28 per 100,000 person-years) and increased more with age in males than in females (Fig. 5; Additional file 1: Table S6). The mortality rate was 35 and 29 per 100,000 person-years in 2002-07 and 2008-13, respectively (Table 5), but no significant age-adjusted annual change was observed (Table 6). Among subgroups of BSI, the mortality rate of HCA-BSI increased in females (7.4% annually), and the mortality rate of BSI from the urinary tract increased in males (11.4% annually). The mortality rate of BSI from pulmonary infection decreased from 10.4 to 5.7 per 100,000 person-years (annually by 9.0% in males). For *Streptococcus pneumoniae* BSI, the mortality rate decreased from 4.8 to 1.9 per 100,000 person-years, with an average annual decrease of 15.0% in males.

**Table 4:** Mortality of bloodstream infection (BSI) stratified by sex in an area of Mid-Norway 2002–2013. Number of deaths and observed and standardized mortality rates, allover and in various subgroups, are shown

	Total			Females			Males		
Group of BSI	*n*	Observed mortality rate^a^ (95% C.I.)	Age and sex standar-dized mortality rate^b^	*n*	Observed mortality rate^a^(95% CI)	Age standar-dized rate^c^	*n*	Observed mortality rate^a^ (95% CI)	Age standar-dized rate^d^
All BSIs	299	35 (31–39)	32	127	29 (24–35)	28	172	40 (35–47)	36
Place of acquisition									
CA-BSI	85	10 (8–12)	9	42	10 (7–13)	9	43	10 (7–14)	9
HCA-BSI	163	19 (16–22)	18	65	15 (12–19)	14	98	23 (19–28)	22
HA-BSI	51	6 (4–8)	6	20	5 (3–7)	4	31	7 (5–10)	7
Infection focus									
Urinary tract	63	7 (6–9)	7	31	7 (5–10)	7	32	8 (5–11)	7
Lungs	75	9 (7–11)	8	25	6 (4–9)	6	50	12 (9–15)	11
Biliary tract	15	2 (1–3)	1	4	1 (0–2)	0.4	11	3 (1–5)	2
Gastrointestinal tract	16	2 (1–3)	2	8	2 (0–4)	2	8	2 (0–3)	2
Skin or soft tissue	34	4 (3–6)	4	12	3 (1–5)	3	22	5 (3–8)	5
Other	28	3 (2–5)	3	14	3 (2–5)	3	14	3 (2–5)	3
Unknown	68	8 (6–10)	7	33	8 (5–11)	7	35	8 (6–11)	8
Microbe group									
Gram-negative BSI	128	15 (12–18)	14	64	15 (11–19)	14	64	15 (12–19)	14
Gram-positive BSI	144	17 (14–20)	15	48	11 (8–15)	11	96	23 (18–27)	20
Polymicrobial or fungal BSI	27	3 (2–5)	3	15	3 (2–6)	3	12	3 (1–5)	3
Microbes (the four most common)									
*Escherichia coli*	59	7 (5–9)	6	28	6 (4–9)	6	31	7 (5–10)	6
*Streptococcus pneumoniae*	31	4 (2–5)	3	10	2 (1–4)	2	21	5 (3–8)	4
*Staphylococcus aureus*	60	7 (5–9)	6	25	6 (4–9)	5	35	8 (6–11)	7
*Klebsiella* spp*.*	20	2 (1–4)	2	12	3 (1–5)	2	8	2 (0.7–4)	2

^a^Deaths within 30 days of BSI episodes per 100,000 person-years (totally 860,630 person-

years in individuals ≥16 years, 426,517 in males, 434,113 in females)

^b^males and females standardized to the age distribution of the male and female population of Norway 2010, respectively

^c^standardized to the age distribution of females in Norway 2010

^d^standardized to the age distribution of males in Norway 2010

**Table 5:** Mortality of bloodstream infection (BSI) in two time periods in an area of Mid-Norway. Number of deaths and observed and standardized mortality rates are shown

	2002–2007			2008–2013		
Group of BSI	*n*	Observed mortality rate^a^ (95% CI)	Age and sex standardized mortality rate^b^	*n*	Observed mortality rate^a^ (95% CI)	Age and sex standardized mortality rate^b^
All BSIs	158	37 (32–44)	35	141	32 (17–53)	29
Place of acquisition						
CA-BSI	50	12 (9–16)	11.0	35	7.9 (5.5–11.0)	7.1
HCA-BSI	77	18 (15–23)	17.0	86	19 (16–24)	17.3
HA-BSI	31	7 (5–11)	7.0	20	4.5 (2.8–7.0)	4.1
Infection focus						
Urinary tract	28	7 (4–10)	6.2	35	7.9 (5.5–11.0)	7.0
Lungs	47	11 (8–15)	10.4	28	6.3 (4.2–9.1)	5.7
Biliary tract	7	1.7 (0.7–3.5)	1.6	8	1.8 (0.78–3.6)	1.5
Gastrointestinal tract	11	2.6 (1.2–4.7)	2.4	5	1.1 (0.37–2.6)	1.0
Skin or soft tissue	16	3.8 (2.2–6.2)	3.5	18	4.1 (2.4–6.4)	3.7
Other	14	3.4 (1.8–5.6)	3.0	14	3.2 (1.7–5.3)	2.9
Unknown	35	8 (6–12)	7.8	33	7.5 (5.1–10.4)	6.8
Microbe group						
Gram-negative BSI	64	15 (12–20)	14.4	64	14 (11–18)	13.0
Gram-positive BSI	81	19 (15–24)	17.9	63	14 (11–18)	12.7
Polymicrobial or fungal BSI	13	3.1 (1.7–5.3)	2.8	14	3.2 (1.7–5.3)	2.8
Microbes (the four most common)^c^						
*Escherichia coli*	31	7 (5–11)	7.1	28	6.3 (4.2–9.1)	5.7
*Streptococcus pneumoniae*	22	5 (3–8)	4.8	9	2.1 (0.9–3.8)	1.9
*Staphylococcus aureus*	30	7 (5–10)	6.7	30	7 (5–10)	6.1
*Klebsiella* spp*.*	8	1.7 (0.7–3.5)	1.6	12	2.7 (1.4–4.7)	2.4

^a^Death within 30 days of BSI episodes per 100,000 person-years (417,682 person-years in 2002–2007; 442,948 person-years in 2008–2013)

^b^age and sex standardized to the population of Norway 2010

^c^mortality in BSI episodes with less common microbes is shown in Additional file 1: Table S7

**Table 6:** Age-adjusted time trends in bloodstream infection (BSI) mortality stratified by sex. BSI rate ratios per calendar year by Poisson regression are shown

	Females		Males	
	Mortality rate ratio (95% CI)	*p-*value	Mortality rate ratio (95% CI)	*p-*value
BSI, all	1.014 (0 .965–1.067)	0.58	0.974 (0.933–1.017)	0.23
Place of acquisition				
Community acquired	0.917 (0.838–1.002)	0.056	0.979 (0.8980–1.068)	0.64
Health care-associated	1.074 (0.999–1.154)	0.052	0.982 (0.928–1.039)	0.53
Hospital acquired	0.998 (0.882–1.129)	0.97	0.915 (0.825–1.015)	0.095
Infection focus				
Urinary tract	0.989 (0.893–1.095)	0.83	1.114 (1.001–1.239)	0.047
Lungs	1.018 (0.908–1.140)	0.76	0.910 (0.838–0.988)	0.024
Biliary tract	0.982 (0.740–1.303)	0.90	1.030 (0.867–1.226)	0.73
Gastrointestinal tract	0.978 (0.800–1.195)	0.83	0.891 (0.724–1.097)	0.28
Skin or soft tissue	1.007 (0.855–1.186)	0.93	0.994 (0.880–1.122)	0.92
Other	0.913 (0.782–1.065)	0.25	1.024 (0.879–1.193)	0.76
Unknown	1.086 (0.981–1.202)	0.11	0.922 (0.836–1.017)	0.10
Microbe group				
Gram-negative BSI	1.023 (0.953–1.098)	0.53	1.011 (0.941–1.085)	0.77
Gram-positive BSI	0.999 (0.921–1.084)	0.99	0.937 (0.884–0.993)	0.027
Polymicrobial or fungal BSI	0.952 (0.829–1.093)	0.49	1.072 (0.911–1.260)	0.40
Microbes (the four most common)				
*Escherichia coli*	1.014 (0.911–1.129)	0.79	0.957 (0.964–1.060)	0.40
*Streptococcus pneumoniae*	0.971 (0.812–1.161)	0.75	0.850 (0.745–0.971)	0.017
*Staphylococcus aureus*	0.985 (0.880–1.103)	0.80	0.972 (0.882–1.070)	0.56
*Klebsiella* spp*.*	1.169 (0.960–1.425)	0.12	1.107 (0.916–1.338)	0.29

### Time trends in case fatality

Allover, the case fatality rate decreased from 17.2% to 13.1% (*p =* 0.014) between 2002–07 and 2008–13, with similar decreases across the three places of acquisition. Among the specific infection foci, the case fatality rate of BSI from pulmonary infection decreased from 28% to 17% (*p =* 0.026) (Additional file 1: Table S8).

### Time trends in blood culture sampling rate

The rate of blood culture sampling increased more than twofold from 2002 to 2013 (2189 to 4605 blood culture sets per 100,000 person-years), and the rate of BSI episodes per 100 blood culture sets decreased from 8.7 in 2002 to 5.6 in 2013 (Additional file 1: Table S3). In the first (2002–07) and second (2008–13) halves of the study period, the average rates of blood culture sampling were 3062 and 3977 sets per 100,000 person-years, and the average rates of BSI episodes per 100 blood culture sets were 7.2 and 6.1, respectively (Additional file 1: Table S9).

## Discussion

The present study provides information on the incidence and mortality of BSI in an area of Mid-Norway, overall, by age and sex, by time period, and for specific subgroups of BSI. The burden of BSI, in terms of both incidence and mortality, increased strongly with age, particularly in males. The incidence increased during the 12-year study period, and the increase was strongest in females, for HCA-BSI, and for urinary tract and Gram-negative BSIs. Over the same period, the mortality remained stable and case fatality rate decreased, possibly because an increased rate of blood culture sampling may have led to improved detection of milder BSI episodes. A shift towards higher proportions of female sex, Gram-negative etiology, and urinary tract site may also have influenced the case fatality rate to some degree. In addition, earlier detection of sepsis and improved treatment may have had impact. Very low, yet slightly increasing rates of microbes with acquired resistance were observed.

To our knowledge, this is the first study estimating the overall burden of BSI in a Norwegian population. Incidence rates of invasive infections with single microbes have been reported [29–33], but no population-based study has focused on BSI as a whole. One previous study described the epidemiology of sepsis in Norway in 1999 [34], and two studies published more than 20 years ago [26, 35] described BSI incidence and mortality related to hospital admissions and bed-days, but did not include population statistics. Strengths of the present study include the prospective registration of BSIs within a well-defined source population. All patients with a BSI acquired outside hospital (CA- and HCA-BSI) in our geographical area were admitted to Levanger Hospital, and the blood cultures were handled at one microbiology laboratory. Some inhabitants in this area will likely have had BSI during stays at St Olav’s Hospital, the closest university hospital, so that the true rate of HA-BSI in our area is slightly higher than is reported in this article. We did not exclude the very small number of episodes that occurred in persons who were visitors to the area, as a similar number of BSIs is likely to have occurred among inhabitants of our area being on travel elsewhere. Standardization for the age and sex distribution is representative for Norway 2010. Use of international population standards could have eased comparison between studies [36, 37], but most studies in this field have used regional or national population standards [4, 8, 10].

The comparison of incidence and mortality rates of BSI between studies is challenging because of differences in the age groups included and in age- and sex distribution of populations [37], differences in the classification of place of acquisition [8, 16, 17, 38], and differences in the definition of incident episodes: first-time vs. total number of BSIs, and different time periods since last BSI required to define a new episode (after 30 days [26], 3 months [6], first episode in another calendar year [36, 37], first episode during the study period [4, 8, 10]). BSI can affect the same individual several times, and the episodes are most often independent of each other. To inform about the total burden of BSI, we chose to estimate the total rate of BSI rather than the first-time episodes only [11]. Compared with our results, incidence rates (166–189 per 100,000 person-years) [6–8] and mortality rates (22 per 100,000 person-years) [6] of BSI are somewhat lower in other studies that also included all BSI episodes. This difference is expected, as these studies also included children, who are generally at low risk of BSI.

An increase in BSI incidence over time have also been reported in Finland (from 147 to 168 per 100,000 person-years from 2004 to 2007) [6] and in Northern Denmark (from 120 to 201 per 100,000 person-years from 1992 to 2006) [8], although a study from Funen, Denmark, [10] of first-time BSI among people ≥16 years of age reported a decrease in BSI incidence from 254 to 199 per 100,000 person-years through 2000 to 2008. Similar to our findings, the increase in BSI incidence was accompanied by a decreasing case fatality rate in Denmark (22.7% to 20.6%) [8], but not in Finland (12.6% to 13.2%) [6]. At the same time, we observed a relatively stable mortality rate of BSI, as has also been reported in Finland [6]. The combination of increased incidence, reduced case fatality, and stable mortality may be explained by improvements in the detection of milder, less fatal BSI episodes. In support of that explanation, we and others (5, 10) have observed increasing rates of blood culture sampling over time, and the number of BSI episodes per 100 blood culture sets decreased with time in our study as was found in Funen, Denmark [10], though not in Northern Denmark or in Finland [5, 8]. Alternatively, the true mortality rate of BSI may have decreased, but the higher detection rate of BSIs may have led to more deaths being attributed to BSI, thus masking a true decline in mortality. In 2007, we updated our local recommendations on sepsis diagnosis and treatment, based on the guidelines of the international Surviving Sepsis Campaign [39], and we performed regular teaching sessions about sepsis for physicians and nurses and implemented standardized observation of patients with suspected sepsis at the wards. Earlier detection and treatment may have improved survival of BSI towards the end of the study period. An in-depth discussion of case fatality rate in the present BSI cohort is given elsewhere [18–22].

The high incidence and mortality of BSI in the older ages in our study, particularly in men, corresponds to what has also been reported by others [4, 6, 40, 41]. While the absolute number of BSIs decreased beyond 85 years, the population beyond this age is progressively smaller and the incidence continued to increase. As the proportion of older people will rise in our part of the world in the decades to come [12, 13], the challenges associated with BSI will escalate.

Compared to our results, HA-BSI accounted for a higher proportion of BSIs (15–58%) in most recent publications, whereas CA-BSI accounted for a lower proportion (18–44%) [1, 42]. An increasing rate of HCA-BSI with time, similar to our results, was also recently reported from Denmark [8], whereas another Danish study found no change with time in HCA-BSI, though reported decreasing incidence rates for both CA-BSI and HA-BSI [10]. In the present study as well as in a Finnish study [6], Gram-negative BSI was most frequent in females whereas Gram-positive BSI predominated in males. During the study period, Gram-negative BSIs increased whereas Gram-positive BSIs decreased. Similar trends were found in Australia [43]. In Finland, however, both Gram-positive and Gram-negative BSIs increased during 2004–2007 [6]. In most other BSI studies as well as in the present study, [4, 8, 11, 38, 44] *E. coli*, *S. pneumoniae*, and *S. aureus* were the three most commonly occurring BSI microbes, accounting for more than one-half of all BSI episodes. The incidence rate of *E. coli* was higher in our study than in other studies (43 per 100,000 person-years reported both from Finland [6] and England [7]), which, however, also included children. One Danish study [10] found a decreasing incidence rate of *E. coli* (70 to 57 per 100,000 person-years). However, another Danish study [8] reported an increasing rate of *E. coli* and reported increased rates of BSI from the urinary tract, similar to the pattern observed by us.

A decreasing occurrence [11, 45–48] and mortality [47] of *S. pneumoniae* is reported in Norway and other countries after the introduction of pneumococcal conjugate vaccines. In Norway, the vaccine was implemented in the immunization program for children in 2006. However, a reduced occurrence of invasive pneumococcal infection is seen also among adults and even among elderly people, due to a herd effect [11, 47]. Furthermore, pneumococcal vaccine is recommended for people >65 years and for those who have undergone an invasive pneumococcal infection. Noteworthy, the decreased rate of *S. pneumoniae* BSI in our study was observed in males but not in females. A possible explanation of this sex difference may be differences in smoking habits, which is a risk factor for invasive pneumococcal disease [49]. The peak prevalence of smoking in Norwegian males occurred 20 years earlier than in females, whose peak prevalence cohort is now in the age group 65–79 years [50].

The incidence of *S. aureus* BSI in our study was similar to a recent study from an area of South-East Norway (27.6 per 100,000 person-years) [32]. The present study as well as others [6, 32] found a higher rate of *S. aureus* BSI in males than in females. The large proportion of HCA infections among the *S. aureus* BSIs was also described in a previous report from our catchment area [21]. *Klebsiella* spp. showed a higher and more increasing (10 to 18 per 100,000 person-years) incidence rate in our study than was found in a Canadian study (7 per 100,000 during 2000–2007) [51]. A nationwide study of invasive *Pseudomonas aeruginosa* infection in Norway 1999–2002 found an incidence rate of 3.2 per 100,000 person-years [31], which was lower than was found in the present study. The incidence rate of candida BSI in our study was slightly lower than what was found in a nationwide study from Norway 2004–2012 (3.9 per 100,000 person-years) [30].

The incidence rate of MRSA in our study was similar to what was found in Copenhagen, Finland, and Western Sweden 2005–08 (<1 per 100,000 person-years) [36], and much lower than what was found in Canada and Australia 2005–08 (7.4 and 4.9 per 100,000 person-years) [36] and in the US and the UK 2006–08 (22 and 3.5 per 100,000 person-years) [52]. Even though ESBL-E has been a rapidly increasing challenge the last 15 years [53, 54], population-based incidence rates for ESBL-E are, so far, not commonly reported. Two Canadian studies found substantially higher and more increasing rates of community onset infections with ESBL-producing microbes (5.5 per 100,000 person-years in 2000–2002 [55], 10.6 per 100,000 person-years some years later [53]) than in the present study.

## Conclusions

Overall, both the incidence and the mortality rates of BSI increased significantly by age, particularly in males. As the proportion of older people increases, geriatric BSIs will be an escalating challenge. The rate of BSI episodes increased through the study period, but the mortality rate was mainly unchanged, and the case fatality rate decreased. A more than twofold increase in the rate of BSI sampling may have contributed to the detection of milder and ultimately less fatal episodes and a shift towards higher proportions of female sex, Gram-negative etiology, and urinary tract site, but earlier detection and improved treatment may have had impact. In pneumococcal BSI, the incidence as well as the mortality decreased in males but not in females. Pneumococcal vaccine probably has contributed, and the difference between sexes is possibly due to sex-specific changes in smoking habits. We observed very low yet slightly increasing rates of microbes with acquired resistance.

## Additional file

Additional file 1: Table S1. Incidence of bloodstream infection in 5-year age groups stratified by sex. **Table S2.** Incidence and mortality of hospital acquired bloodstream infection. **Table S3.** Incidence of bloodstream infection and blood culture sampling by calendar year. **Table S4.** Incidence and mortality of hospital acquired bloodstream infection in 2002–2007 compared to 2008–2013. **Table S5.** Less common bloodstream infection microbes in two time periods in an area of Mid-Norway. **Table S6.** Mortality of bloodstream infection in 5-year age groups stratified by sex. **Table S7.** Mortality of bloodstream infection with less common microbes. **Table S8.** Case fatality rate of bloodstream infection in two time periods. **Table S9.** Rate of blood culture sampling in 2002–2007 compared to 2008–2013. (DOCX 60 kb)

## Abbreviations

ADR: Acquired drug resistance
BSI: Bloodstream infection
CA: Community acquired
ESBL-E: Extended-spectrum beta-lactamase producing *Enterobacteriaceae*
HA: Hospital acquired
HCA: Healthcare-associated
MRSA: Methicillin-resistant *Staphylococcus aureus*
PNSP: Penicillin-non-susceptible pneumococci
