# Supplementary material for: Burden of bloodstream infection in an area of Mid-Norway 2002-2013: a prospective population-based observational study
Source: BMC Infect Dis. 2017 Mar 11;17:205. doi: 10.1186/s12879-017-2291-2 (PMC5346205; doi:10.1186/s12879-017-2291-2)
Supplement: Additional file 1: Table S1. — Incidence of bloodstream infection in 5-year age groups stratified by sex. Table S2. Incidence and mortality of hospital acquired bloodstream infection. Table S3. Incidence of bloodstream infection and blood culture sampling by calendar year. Table S4. Incidence and mortality of hospital acquired bloodstream infection in 2002–2007 compared to 2008–2013. Table S5. Less common bloodstream infection microbes in two time periods in an area of Mid-Norway. Table S6. Mortality of bloodstream infection in 5-year age groups stratified by sex. Table S7. Mortality of bloodstream infection with less common microbes. Table S8. Case fatality rate of bloodstream infection in two time periods. Table S9. Rate of blood culture sampling in 2002–2007 compared to 2008–2013. (DOCX 60 kb) [file 12879_2017_2291_MOESM1_ESM.docx]

Additional file 1

**Burden of bloodstream infection in an area of Mid-Norway 2002-2013.**

Mehl et al.

**Table S1** Incidence of bloodstream infection (BSI) in 5-year age groups stratified by sex. Number of BSI episodes per 100,000 person-years is shown

|  | Males |  | Females |  |
| --- | --- | --- | --- | --- |
|  |  | BSI rate |  | BSI rate |
|  | n | n/100,000 person-years (95% CI) | n | n/100,000 person-years (95% CI) |
| Age group (years) |  |  |  |  |
| 16-19 | 11 | 35 (18-63) | 7 | 23 (9-48) |
| 20-24 | 7 | 20 (8-42) | 8 | 24 (11-48) |
| 25-29 | 11 | 38 (19-68) | 20 | 72 (44-111) |
| 30-34 | 17 | 53 (31-85) | 12 | 38 (20-67) |
| 35-39 | 15 | 40 (23-66) | 25 | 69 (44-101) |
| 40-44 | 19 | 48 (29-75) | 20 | 53 (33-82) |
| 50-54 | 45 | 120 (88-161) | 40 | 112 (80-115) |
| 55-59 | 50 | 136 (101-180) | 63 | 176 (136-226) |
| 60-64 | 93 | 288 (232-353) | 62 | 196 (151-252) |
| 65-69 | 95 | 387 (313-473) | 86 | 342 (274-423) |
| 70-74 | 124 | 664 (552-792) | 94 | 463 (374-566) |
| 75-79 | 167 | 1085 (926-1262) | 125 | 670 (558-798) |
| 80-84 | 173 | 1521 (1303-1766) | 174 | 1063 (911-1233) |
| 85-89 | 133 | 2225 (1863-2637) | 130 | 1189 (993-1411) |
| 90-94 | 40 | 2109 (1506-2871) | 56 | 1220 (921-1584) |
| 95-99 | 8 | 2827 (1220-5570) | 12 | 1037 (536-1812) |

**Table S2** Incidence and mortality of hospital acquired bloodstream infection (HA-BSI). HA-BSI episodes and deaths within 30 days of episodes per 100,000 hospital bed-days by calendar year and overall are shown

| Calendar year | Hospital bed-days | HA-BSI episodes | HA-BSI episodes per 100 000 hospital bed-days | Deaths within 30 days | Mortality per 100 000 hospital bed-days |
| --- | --- | --- | --- | --- | --- |
| 2002 | 59,906 | 24 | 40.1 (25.7-59.6) | 4 | 6.7 (1.8-17.1) |
| 2003 | 62,114 | 15 | 24.1 (13.5-39.8) | 4 | 6.4 (1.8-16.5) |
| 2004 | 63,810 | 26 | 40.7 (26.6-59.7) | 2 | 3.1 (0.38-11.3) |
| 2005 | 61,837 | 25 | 40.4 (26.2-59.7) | 7 | 11.3 (4.6-23.3) |
| 2006 | 61,874 | 21 | 33.9 (21.1-51.9) | 6 | 9.7 (3.6-21.1) |
| 2007 | 60,577 | 23 | 38.0 (24.1-57.0) | 8 | 13.2 (5.7-26.0) |
| 2008 | 62,222 | 15 | 24.1 (13.5-39.8) | 1 | 1.6 (0.41-9.0) |
| 2009 | 56,655 | 22 | 38.8 (24.3-58.8) | 4 | 7.1 (1.9-18.1) |
| 2010 | 56,601 | 25 | 44.2 (28.6-65.2) | 6 | 10.6 (3.9-23.1) |
| 2011 | 58,642 | 27 | 46.0 (30.3-67.0) | 4 | 6.8 (1.9-17.4) |
| 2012 | 59,113 | 22 | 37.2 (23.3-56.4) | 3 | 5.1 (1.1-14.8) |
| 2013 | 58,731 | 29 | 49.4 (33.1-70.9) | 2 | 3.4 (0.41-12.3) |
| Total | 722,082 | 274 | 37.9 (33.6-42.7) | 51 | 7.1 (5.3-9.3) |

**Table S3** Incidence of bloodstream infection (BSI) and blood culture sampling by calendar year. The observed incidence of BSI episodes, the rate of blood culture sampling, and the number of BSI episodes per 100 blood culture sets are shown.

| Calendar year | Population* | BSI episodes | BSI episodes per 100,000 person-years | Blood culture sets | Blood culture sets per 100,000 person-years | BSI episodes per 100 blood culture sets |
| --- | --- | --- | --- | --- | --- | --- |
| 2002 | 68,491 | 130 | 190 (159-225) | 1499 | 2189 (2079-2302) | 8.7 (7.2-10.3) |
| 2003 | 68,746 | 131 | 191 (159-226) | 1803 | 2623 (2503-2747) | 7.3 (6.1-8.6) |
| 2004 | 69,236 | 163 | 235 (201-274) | 2075 | 2997 (2869-3129) | 7.9 (6.7-9.2) |
| 2005 | 69,883 | 158 | 226 (192-264) | 2340 | 3348 (3214-3487) | 6.8 (5.7-7.9) |
| 2006 | 70,363 | 163 | 232 (197-270) | 2539 | 3608 (3469-3752) | 6.4 (5.5-7.5) |
| 2007 | 70,963 | 176 | 248 (213-287) | 2535 | 3572 (3435-3714) | 6.9 (6.0-8.0) |
| 2008 | 71,612 | 143 | 200 (168-235) | 2499 | 3490 (3354-3629) | 5.7 (4.8-6.7) |
| 2009 | 73,357 | 156 | 213 (181-249) | 2195 | 2992 (2868-3120) | 7.1 (6.0-8.3) |
| 2010 | 73,225 | 180 | 246 (211-284) | 2782 | 3799 (3659-3943) | 6.5 (5.6-7.5) |
| 2011 | 73,976 | 206 | 278 (242-319) | 3348 | 4526 (4374-4682) | 6.2 (5.3-7.1) |
| 2012 | 74,920 | 194 | 259 (224-298) | 3299 | 4403 (4254-4556) | 5.9 (5.1-6.8) |
| 2013 | 75,858 | 195 | 257 (222-296) | 3493 | 4605 (4453-4760159) | 5.6 (4.8-6.4) |

*≥16 years

**Table S4** Incidence and mortality of hospital acquired bloodstream infection (HA-BSI) in 2002-2007 compared to 2008-2013 (the denominator is hospital bed-days)

|  | 2002-2007 | 2008-2013 |
| --- | --- | --- |
| HA-BSI episodes | 134 | 140 |
| Hospital bed-days | 370,118 | 351,964 |
| HA-BSI episodes per 100,000 hospital bed-days | 36.2 (30.3-42.9) | 39.8 (33.5-46.9) |
| Deaths within 30 days in HA-BSI episodes | 31 | 20 |
| HA-BSI mortality per 100,000 hospital bed-days | 8.4 (5.7-11.9) | 5.7 (3.5-8.8) |

**Table S5** Less common bloodstream infection (BSI) microbes in two time periods in an area of Mid-Norway. Numbers of episodes and observed and standardized incidence rates of microbes not listed in Table 2 are shown. Microbes with inherent or acquired resistance are included

|  | 2002-2007 | | | 2008-2013 | | |
| --- | --- | --- | --- | --- | --- | --- |
| **Other BSI microbes#** | n | Observed  incidence rate*  (95% CI) | Age and sex  standardized  incidence  rate§ | n | Observed  incidence rate*  (95% CI) | Age and sex  standardized  incidence  rate§ |
| Other *Enterobacteriaceae* | 57 | 13.7 (10.3-17.7) | 13 | 64 | 14.4 (11.1-18.4) | 14 |
| Beta-hemolytic streptococci |  |  |  |  |  |  |
| Group A | 21 | 5.0 (3.1-7.7) | 4.7 | 15 | 3.4 (1.8-5.6) | 3.1 |
| Group B | 18 | 4.3 (2.6-6.8) | 4.0 | 18 | 4.1 (2.4-6.4) | 3.8 |
| Group C or G | 16 | 3.8 (2.2-6.2) | 3.5 | 16 | 3.6 (2.1-5.9) | 3.1 |
| *Enterococcus* spp*.* | 38 | 9.1 (6.4-12.5) | 8.7 | 51 | 11.5 (8.6-15.1) | 10.6 |
| *E. faecalis* | 31 | 7.4 (5.0-10.5) | 7.1 | 45 | 10.2 (7.4-13.6) | 9.3 |
| *E. faecium* | 3 | 0.7 (0.15-3.1) | 0.6 | 5 | 1.1 (0.4-2.6) | 1.1 |
| Anaerobic bacteria | 25 | 6.0 (3.9-8.8) | 5.3 | 31 | 7.0 (4.8-9.9) | 6.5 |
| Coagulase-negative staphylococci | 28 | 6.7 (4.5-9.7) | 6.3 | 26 | 5.9 (3.8-8.6) | 5.3 |
| *Haemophilus influenzae* | 11 | 2.6 (1.3-4.7) | 2.4 | 6 | 1.4 (0.5-3.0) | 1.3 |
| *Neisseria meningitidis* | 6 | 1.4 (0.5-3.1) | 1.5 | 3 | 0.7 (1-2.0) | 0.7 |
|  |  |  |  |  |  |  |
| **Microbes with inherent resistance** |  |  |  |  |  |  |
| *Pseudomonas aeruginosa* | 33 | 7.9 (5.4-11.1) | 6.7 | 29 | 6.5 (4.4-9.4) | 5.8 |
| *Acinetobacter* spp*.* | 2 | 0.5 (0.06-1.7) | 0.5 | 5 | 1.1 (0.4-2.6) | 1.0 |
| *Stenotrophomonas maltophilia* | 0 | 0 | 0 | 2 | 0.5 (0.1-1.6) | 0.4 |
| *Elisabethkingia meningo-*  *septica* | 0 | 0 | 0 | 1 | 0.2 (0.06-1.2) | 0.2 |
| **Microbes with acquired**  **resistance** |  |  |  |  |  |  |
| MRSA | 0 | 0 | 0 | 1 | 0.2 (0.06-1.2) | 0.2 |
| ESBL-E | 3 | 0.7 (0.15-2.1) | 0.6 | 7 | 1.6 (0.6-3.3) | 1.4 |
| PNSP | 0 | 0 | 0 | 2 | 0.5 (0.1-1.6) | 0.4 |
| **Fungi** |  |  |  |  |  |  |
| *Candida* spp*.* | 6 | 1.4 (0.5-3.1) | 1.4 | 11 | 2.5 (1.2-4.4) | 2.3 |

#microbes not noted in Table 2

*BSI episodes per 100,000 person-years (417,682 person-years in 2002-2007; 442,948 person-years in 2008-2013)

§age and sex standardized to the population of Norway 2010

ESBL-E, extended spectrum beta-lactamase producing *Enterobacteriaceae;* MRSA, methicillin-resistant *Staphylococcus aureus*; PNSP, penicillin non-susceptible pneumococci

**Table S6** Mortality of bloodstream infection (BSI) in 5-year age groups stratified by sex. The table shows the number of deaths within 30 days of BSI episodes per 100,000 person-years

| **Age group** | **Males** | | **Females** | |
| --- | --- | --- | --- | --- |
|  | n | BSI mortality rate  n/100,000 person-years (95% CI) | n | BSI mortality rate  n/100,000 person-years (95% CI) |
|  |  |  |  |  |
| 16-19 | 0 | 0 | 1 | 3.3 (0.08-18.6) |
| 20-24 | 0 | 0 | 0 | 0 |
| 25-29 | 1 | 3.4 (0.09-19.2) | 0 | 0 |
| 30-34 | 0 | 0 | 0 | 0 |
| 35-39 | 0 | 0 | 0 | 0 |
| 40-44 | 1 | 2.5 (0.06-14.2) | 0 | 0 |
| 45-49 | 1 | 2.6 (0.07-14.6) | 1 | 2.7 (0.07-14.8) |
| 50-54 | 7 | 18.7 (7.5-38.6) | 3 | 8.4 (1.7-24.4) |
| 55-59 | 7 | 19.0 (7.6-39.1) | 6 | 16.8 (6.2-36.5) |
| 60-64 | 12 | 37.1 (19.2-64.9) | 4 | 12.7 (3.5-32.4) |
| 65-69 | 18 | 73.3 (43.4-115.8) | 8 | 31.8 (13.7-62.7) |
| 70-74 | 20 | 107.1 (65.4-165.4) | 10 | 49.2 (23.6-90.5) |
| 75-79 | 27 | 175.3 (115.6-255.2) | 27 | 144.7 (95.4-210.6) |
| 80-84 | 32 | 281.4 (192.5-397.2) | 28 | 171.0 (113.6-247.1) |
| 85-89 | 28 | 468.4 (311.2-677.0) | 22 | 201.1 (126.1-304.5) |
| 90-94 | 13 | 685.3 (365.9-1171.9) | 12 | 261.4 (135.1-456.6) |
| 95-99 | 5 | 1766.8 (573.7-412.3) | 5 | 432.2 (140.3-1008.5) |

**Table S7** Mortality of bloodstream infection (BSI) with less common microbes. Number of deaths and observed and standardized mortality rates in 2002-2007 and 2008-2013 are shown

|  | 2002-2007 | | | 2008-2013 | | |
| --- | --- | --- | --- | --- | --- | --- |
| **Other BSI microbes#** | n | Observed  mortality rate*  (95% CI) | Age and sex  standardized  mortality  rate§ | n | Observed  mortality rate*  (95% CI) | Age and sex  standardized  mortality  rate§ |
| Other *Enterobacteriaceae* | 10 | 2.4 (1.2-4.4) | 2.1 | 7 | 1.6 (0.6-3.3) | 1.4 |
| Beta-hemolytic streptococci | 9 | 2.2 (1.0-4.1) | 2.0 | 8 | 1.8 (0.8-3.6) | 1.6 |
| *Enterococcus* spp*.* | 5 | 1.2 (0.4-2.8) | 1.2 | 5 | 1.1 (0.4-2.6) | 1.0 |
| Anaerobic bacteria | 5 | 1.2 (0.4-2.8) | 1.1 | 3 | 0.7 (0.1-2.0) | 0.6 |
| *Pseudomonas* spp*.* | 6 | 1.4 (0.5-3.1) | 1.3 | 5 | 1.1 (0.4-2.6) | 1.0 |
| Coagulase-negative staphylococci | 4 | 1.0 (0.3-2.5) | 0.8 | 4 | 0.9 (0.25-2.3) | 0.8 |
| *Haemophilus influenzae* | 3 | 0.7 (0.1-2.1) | 0.7 | 1 | 0.2 (0.006-1.3) | 0.2 |
| *Neisseria meningitidis* | 2 | 0.5 (0.06-1.7) | 0.5 | 0 | 0 | 0 |
|  |  |  |  |  |  |  |
| **Fungi** |  |  |  |  |  |  |
| *Candida* spp*.* | 3 | 0.7 (0.1-2.1) | 0.7 | 6 | 1.4 (0.5-3.0) | 1.2 |

#mortality in BSI episodes with the four most common microbes is shown in Table 4

*Death within 30 days of BSI episodes per 100,000 person-years (417,682 person-years in 2002-2007; 442,948 person-years in 2008-2013)

§age and sex standardized to the population of Norway 2010

**Table S8** Case fatality rate (CFR) of bloodstream infection (BSI) in two time periods. Number of deaths and observed CFR, overall and in subgroups, are shown

|  | Total | | 2002-2007 | | 2008-2013 | | p-value |
| --- | --- | --- | --- | --- | --- | --- | --- |
|  | No. | CFR (95% CI) | No. | CFR (95% CI) | No. | CFR (95% CI) |  |
| **All BSI episodes** | 299 | 15.0 (13.5-16.6) | 158 | 17.2 (14.9-19.7) | 141 | 13.1 (11.2-15.3) | 0.014 |
|  |  |  |  |  |  |  |  |
| **Place of acquisition** |  |  |  |  |  |  |  |
| CA-BSI | 85 | 9.1 (7.4-11.1) | 50 | 10.6 (8.1-13.7) | 35 | 7.6 (5.5-10.3) | 0.11 |
| HCA-BSI | 163 | 20.7 (18.0-23.7) | 77 | 24.4 (20.0-29.4) | 86 | 18.3 (15.0-22.0) | 0.040 |
| HA-BSI | 51 | 18.6 (14.4-23.6) | 31 | 23.1 (16.8-31.0) | 20 | 14.3 (9.4-21.0) | 0.064 |
|  |  |  |  |  |  |  |  |
| **Infection focus** |  |  |  |  |  |  |  |
| Urinary tract | 63 | 8.3 (6.6-10.6) | 28 | 8.9 (6.2-12.5) | 35 | 8.0 (5.8-11.0) | 0.69 |
| Lungs | 75 | 22.7 (18.5-27.5) | 47 | 27.8 (21.6-35.0) | 28 | 17.3 (12.2-23.8) | 0.026 |
| Biliary tract | 15 | 6.8 (4.2-10.9) | 7 | 6.6 (3.2-13.0) | 8 | 7.0 (3.6-13.2) | >0.99 |
| Gastrointestinal tract | 16 | 15.8 (10.0-24.2) | 11 | 23.4 (13.6-37.2) | 5 | 9.3 (4.0-19.9) | 0.061 |
| Skin or soft tissue | 34 | 23.8 (17.5-31.4) | 16 | 19.8 (12.5-29.7) | 18 | 29.0 (19.2-41.3) | 0.24 |
| Other | 28 | 11.3 (8.0-15.9) | 14 | 13.0 (7.9-20.6) | 14 | 10.1 (6.1-16.2) | 0.55 |
| Unknown | 68 | 33.8 (27.6-40.6) | 35 | 37.2 (28.1-47.3) | 33 | 30.8 (22.9-40.1) | 0.327 |
|  |  |  |  |  |  |  |  |
| **Microbe group** |  |  |  |  |  |  |  |
| Gram-negative BSI | 128 | 11.3 (9.6-13.3) | 64 | 12.8 (10.2-16.0) | 64 | 10.1 (8.0-12.7) | 0.16 |
| Gram-positive BSI | 144 | 18.5 (16.0-21.4) | 81 | 21.0 (17.2-25.3) | 63 | 16.1 (12.8-20.1) | 0.096 |
| Polymicrobial or fungal BSI | 27 | 31.8 (22.8-42.3) | 13 | 36.1 (22.4-52.4) | 14 | 28.6 (17.8-42.4) | 0.49 |
|  |  |  |  |  |  |  |  |
| **Microbes** |  |  |  |  |  |  |  |
| *Escherichia coli* | 59 | 8.6 (6.7-10.9) | 31 | 10.2 (7.3-14.2) | 28 | 7.3 (5.1-10.4) | 0.22 |
| *Streptococcus pneumoniae* | 31 | 13.7 (9.8-18.8) | 22 | 17.3 (11.7-24.8) | 9 | 9.1 (4.9-16.4) | 0.082 |
| *Staphylococcus aureus* | 60 | 27.5 (22.0-33.8) | 30 | 30.0 (21.9-39.6) | 30 | 25.4 (18.4-34.0) | 0.53 |
| *Klebsiella* spp*.* | 20 | 14.8 (9.8-21.8) | 8 | 17.0 (8.9-30.1) | 12 | 13.6 (8.0-22.3) | 0.62 |
| Other *Entero-bacteriaceae* | 16 | 13.2 (8.3-20.4) | 9 | 15.8 (8.5-27.4 | 7 | 10.9 (5.4-20.9) | 0.59 |
| Beta-hemolytic streptococci | 17 | 16.3 (10.5-24.6) | 9 | 16.4 (8.9-28.3) | 8 | 16.3 (8.5-29.0) | >0.99 |
| *Enterococcus* spp*.* | 10 | 11.2 (6.2-19.5) | 5 | 13.2 (5.8-27.3) | 5 | 9.8 (4.3-21.0) | 0.74 |
| Anaerobic bacteria | 8 | 14.3 (7.4-25.7) | 5 | 20.0 (8.9-39.1) | 3 | 9.7 (3.3-24.9) | 0.45 |
| *Pseudomonas* spp*.* | 11 | 19.0 (10.9-30.9) | 6 | 20.0 (9.5-37.3) | 5 | 17.9 (7.9-35.6) | >0.99 |
| Coagulase-negative staphylococci | 8 | 14.8 (7.7-26.6) | 4 | 14.3 (5.7-31.5) | 4 | 15.4 (6.1-33.5) | >0.99 |

**Table S9** Rate of blood culture sampling in 2002-2007 compared to 2008-2013

|  | 2002-2007 | 2008-2013 |
| --- | --- | --- |
| Person-years | 417,682 | 442,948 |
| Blood culture sets | 12,791 | 17,616 |
| Blood culture sets per 100,000 person-years | 3062 | 3977 |
|  |  |  |
| BSI episodes | 921 | 1074 |
| BSI episodes per 100 blood culture sets | 7.2 (6.7-7.7) | 6.1 (5.7-6.5) |
